# Genomic Characterization of a Novel Alphacoronavirus Isolated from Bats, Korea, 2020

**DOI:** 10.3390/v13102041

**Published:** 2021-10-11

**Authors:** Hai-Quynh Do, Van-Giap Nguyen, Chul-Un Chung, Yong-Shin Jeon, Sook Shin, Kuem-Chan Jang, Le Bich Hang Pham, Aeri Kong, Cheong-Ung Kim, Yong-Ho Park, Bong-Kyun Park, Hee-Chun Chung

**Affiliations:** 1 Virology Lab, Department of Veterinary Medicine, College of Veterinary Medicine and Research Institute for Veterinary Science, Seoul National University, Seoul 08826, Korea; quynhdohai@gmail.com; 2 Department of Veterinary Microbiology and Infectious Diseases, Faculty of Veterinary Medicine, Vietnam National University of Agriculture, Hanoi 100000, Vietnam; nvgiap@vnua.edu.vn; 3 Department of Life Science, Dongguk University, Gyeongju 38066, Korea; bigboss369@naver.com; 4 Noah Biotech Research Unit, Noah Biotech Co. Ltd, Suwon 16612, Korea; baram8388@snu.ac.kr (S.S.); xmfkwp7@naver.com (K.-C.J.); yhp@snu.ac.kr (Y.-H.P.); 5 Institute of Genome Research, Vietnam Academy of Science and Technology, Hanoi 100000, Vietnam; plbhang@igr.ac.vn; 6 Department of Medical Science, University of California, Los Angeles, CA 90095, USA; aerikong95@gmail.com; 7 Department of Veterinary Medicine Microbology Lab, College of Veterinary Medicine and Research Institute for Veterinary Science, Seoul National University, Seoul 08826, Korea; kcu705@snu.ac.kr

**Keywords:** *Alphacoronavirus*, novel species, bat, Korea

## Abstract

Coronavirus, an important zoonotic disease, raises concerns of future pandemics. The bat is considered a source of noticeable viruses resulting in human and livestock infections, especially the coronavirus. Therefore, surveillance and genetic analysis of coronaviruses in bats are essential in order to prevent the risk of future diseases. In this study, the genome of HCQD-2020, a novel alphacoronavirus detected in a bat (*Eptesicus serotinus*), was assembled and described using next-generation sequencing and bioinformatics analysis. The comparison of the whole-genome sequence and the conserved amino acid sequence of replicated proteins revealed that the new strain was distantly related with other known species in the *Alphacoronavirus* genus. Phylogenetic construction indicated that this strain formed a separated branch with other species, suggesting a new species of *Alphacoronavirus*. Additionally, *in silico* prediction also revealed the risk of cross-species infection of this strain, especially in the order Artiodactyla. In summary, this study provided the genetic characteristics of a possible new species belonging to *Alphacoronavirus.*

## 1. Introduction

Coronavirus, a group of enveloped, positive single-stranded RNA of approximately 30 kb in length, belongs to the subfamily *Orthocoronavirinae,* family *Coronaviridae*, and is classified into four genera: *Alphacoronavirus*, *Betacoronavirus*, *Gammacoronavirus,* and *Deltacoronavirus* [1]. Alphacoronavirus has been recognized as the causative agent of mild respiratory syndromes in humans, such as the human coronavirus (HuCoV) NL63 and HuCoV 229E, and serious respiratory diseases in livestock [2]. Transmissible gastroenteritis coronavirus (TGEV), porcine epidemic diarrhea virus (PEDV), and recently, porcine enteric alphacoronavirus (PEAV) are the major viruses responsible for most of the pandemics in pigs, causing huge economic losses [3,4]. Betacoronavirus causes several deadly diseases in humans such as severe acute respiratory syndrome (SARS), Middle East respiratory syndrome (MERS), and Coronavirus Disease-19 (COVID-19) [2]. Most of these diseases have originated from wild animals.

Due to their large genome size, high mutation rates, and recombination between homologous RNA regions, coronaviruses are considered as one of the most diversified viruses [5]. The extreme diversity of coronaviruses has been observed in rodents and bats worldwide [6,7,8]. In detail, almost all corona positive samples collected in Asia, Africa, and North America were bats which account for 91/100 different taxonomic units [6]. Three different coronavirus groups were observed in 16 rodent species belonging to seven different genera. In addition to the two well-known reservoirs mentioned above, coronavirus was also prevalent in rabbits and hedgehogs in France [7]. Although the study focused on the highly conserved region of Rna-dependent RNA polymerase (RdRp) encoding genes, many potentially new *Coronavirus* species have been detected in wild animals around the world [9,10,11]. Recently, six novel species/variants were obtained in rodents [12] and bats [11] in China. Bat species’ richness, for instance, was correlated with the diversity of coronavirus [6]. The diversity of coronaviruses and high recombinant rates, on the other hand, increased the risk of host switch and ecological niche adaptations [13]. The sequence analysis of multiple complete genomes of camel coronavirus revealed a trace of rabbit coronavirus and rodent coronavirus [14]. Additionally, viral quasispecies followed by selection might have played an important role for coronavirus during the new host adaptation [15].

Bats are unique mammals (with distinct characteristics such as a long lifespan, being the only mammal capable of actually flying, and a gregarious nature), and they are widely distributed worldwide. As a result, they come into easy contact with other animals. Bats are important original reservoirs for a vast number of zoonotic viruses, which cause serious infections in humans and livestock [16]. Several strains of *Alphacoronavirus* and *Betacoronavirus* detected in bats have been known to induce diseases such as COVID-19, responsible for the current pandemic, as well as many serious infectious diseases in livestock [17,18]. In fact, more than one-third of viruses detected in bats belong to the *Coronaviridae* family [19]. The prevalence of coronaviruses in bats was 15.2% in Korea [20] and 6.8% in China [11]. Corona infection rates of bats were estimated at 1.7% in Gabon [21] and 3.7% in Brazil [22]. Host restriction of the bat coronavirus is still under debate. Some studies suggest that the coronavirus might be restricted to the bat genus or below level [23,24]. A genetic analysis of Rdrp encoding fragments of bat coronavirus in Northern Germany indicated that closely related coronavirus strains were more likely associated with the bat species than with the location of the sampling sites [25]. On the other hand, others supported the wide host range of coronavirus groups [5,9]. BatCoV HKU10 strains detected in different bat species shared a highly similar sequence throughout the genome, except the genes encoding for the spike protein, which contributed to the new host adaptation [26]. Similar phenomena were also observed in the cases of SARS-CoV2 infections in humans and SARS detected in bats [27].

Therefore, it is necessary to continue active surveillance and genetic analysis of newly detected coronaviruses in bats. In Korea, previous studies based on partial RdRp indicated that bats contained a diversity of alpha- and beta-coronaviruses [20,28]. However, a genomic-based approach provides more in-depth analysis into the diversity of bat coronaviruses in terms of genetic variation. As a result, we describe the complete genomic characteristics of HCQD-2020, a novel *Alphacoronavirus* species isolated from a Korean bat species, *Eptesicus serotinus*.

## 2. Materials and Methods

### 2.1. Sampling, RNAExtraction and RT-PCR

From July to September 2020, six carcasses of different microbat species (*Eptesicus serotinus*, *Myotis petax*, *M*. *ikonnibovi*, and *Pipistrellus abramus*) were collected from Kangwon and Gyeongbuk provinces (Table S1). Samples were kept in ice packages and then transferred to the College of Veterinary Medicine, Seoul National University. Organs of the bat carcasses (lung, intestine, and liver) were homogenized in 1 ml of Dulbecco’s Modified Eagle Medium (DMEM) followed by three cycles of freeze–thaw procedure. An amount of 150 µL of this homogenized solution was used for RNA extraction using a DNA/RNA extraction kit (Intron Biotech, Gyeonggi-do, Korea) according to the manufacturer’s protocol. Previously published pancoronavirus primers [29] were applied for screening the presence of coronavirus in the bat samples (Table S2). RT-PCR reactions using the TOPScript One-step RT-PCR kit (Enzynomics, Daejeon, Korea) were performed under the condition of initial heating of 50 °C for 30 min, 95 °C for 10 min; followed by 40 cycles of 95 °C for 30 s, 55 °C for 30 s and 72 °C for 30 s; and a final elongation step at 72 °C for 7 min. Corrected bands were purified by gel extraction followed by directed DNA sequencing.

### 2.2. Whole-Genome Sequencing, Genome Assembly, and Annotation

To prepare RNA samples for next-generation sequencing (NGS), 0.5 mL of the homogenized solution was treated with 10 µL of RNase (4 mg/mL) (Biosesang, Gyeonggi-do, Korea) and 10 µL of DNase (10 U/µL) (Promega, Madison, WI, USA) for 30 min. The nuclease-treated solution was filtered through a 0.2 µm filter (Sartorius, Goettingen, Germany). Finally, particle-associated RNA was extracted as described above. The RNA sample was sent to Macrogen for NGS using a library of 346 bp in size.

Raw data of 101 bp pair-end sequencing was filtered to remove the low-quality base calling by FastQC using the recommended parameters. Filtered reads were assembled *de novo* using SPAdes software [30]. A scaffold related to coronavirus was detected by Blastn by comparing with the coronavirus database. Next, the 3′-end sequencing was performed as described elsewhere [31].

Whole genome sequence of the novel coronavirus was annotated by the Z-curve tools [32]. Putative structural and non-structural proteins were validated by the Blastp method. Functional domains of the proteins were analyzed by Interpro (https://www.ebi.ac.uk/interpro/search/sequence-search accessed on 4 April 2021) [33] using the following databases: CATH-gene3D, CDD, MobiDB, HAMAP, PANTHER, Pfam, PIRSF, PRINTS, ProDom, PROSITE, SFLD, SMART, SUPERFAMILY, and TIGRFAMs. RNA structural elements were scanned with the Rfam database (https://rfam.xfam.org/ accessed on 5 April 2021) [34].

### 2.3. Sequence Alignment and Phylogenetic Construction

Recombinant events were commonly detected and continuously played roles in the evolution of coronaviruses [35,36]. For the purpose of classification with previously known, well-defined viruses of *Alphacoronavirus* genus, this study did not perform recombination analysis prior to phylogenetic reconstructions. All phylogenetic trees were inferred based on the whole genome, structural and nonstructural protein-encoding genes rather than the genomic fragment in between the predicted breakpoints.

The obtained genome, structural- and nonstructural- protein-encoding genes were aligned with those of the representative species belonging to the *Alphacoronavirus* genus (Supplement data) by MAFFT algorithm [37]. Phylogeny trees were constructed using Iqtree2 [38] using the best-fit substitution model, automatically selected by option “-m MFP” [39]. Statistical support was obtained by performing ultrafast bootstrap approximation [40]. The constructed trees have been displayed by FigTree v1.4.4 (https://github.com/rambaut/figtree/ accessed on 9 April 2021).

### 2.4. Potential Host Prediction

In order to investigate the cross-infection of this strain, an online web tool (available at http://host-predict.cvr.gla.ac.uk/ accessed on 2 May 2021) was used to predict the potential reservoir host [41]. A model combining genomic biases and phylogenetic neighborhood was applied in this study for greater accuracy. In detail, the coding sequence of all putative genes and the whole genome of HCQD-2020 strain were used for prediction. The results are represented as a box-plot graph displaying the min, 25th percentile, median, 75th percentile, and max probability scores of each group of reservoir hosts. The higher the score, the more significant the probability that a group of hosts acted as a reservoir.

## 3. Results

### 3.1. Coronavirus Detection in Bat Samples

To examine the presence of coronavirus in the bat samples collected in this study, organ samples from each bat including the lungs, intestine, and liver were applied for RNA extraction followed by RT-PCR using the pan-CoV primers. Of these, only the intestinal sample from *E. serotinus* collected from Gyeongbuk exhibited a single band of 440 bp as expected. All other samples were negative with coronavirus. Therefore, we extracted this band for Sanger sequencing. A phylogenetic analysis indicated that this isolate belonged to the *Alphacoronavirus* genus (Figure S1).

### 3.2. Whole-Genome Assembly and Annotation

**Figure 1:**
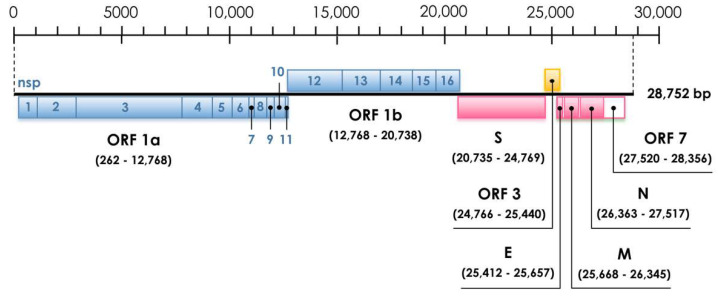
Genome organization and whole-genome phylogenetic analysis of novel *Alphacoronavirus* strain HCQD-2020. This strain contained seven open reading frames (ORFs): ORF1a, ORF1b (light blue box as nonstructural proteins); S, E, M, N (pink box as structural proteins); ORF3 (orange box as accessory protein) and unknown function ORF7 (white box).

Whole-genome sequencing using the Illumina platform was carried out to further analyze the genomic characteristics of the isolate detected in this study. A total of 7.2 Gbps with percentages of high-quality base calling of 98.74% and 96.46% for Q20 and Q30 was obtained. A near complete genome (28,752 nucleotides excluding the poly-A tail with the average depth of 30X) of the alphacoronavirus strain HCQD-2020 (GenBank accession number: MW924112) was obtained and annotated. Sequence annotation showed that this strain contains seven common open reading frames (ORFs) in the typical order 5′-UTR-ORF1ab-S-ORF3-E-M-N-ORF7-3′-UTR (Figure 1). Hexanucleotide transcriptional regulatory sequences (TRSs) required for the transcription of complete and subgenomic RNA were also identified (Table 1). Additionally, the putative signal sequences, including a partial 5′-UTR, a 3′-UTR, and a coronavirus frameshifting stimulation element, conserved the slippery sequence (Table 2). The characteristics of putative nonstructural proteins (NSP) 1–16 are described in Table 3. The appearance of a small ORF (or ORFs), with unknown function, (normally named as ORF7) downstream of the nucleocapsid encoding gene has been reported in some other species belonging to this genus [42]. Nevertheless, in this study, neither Blastn nor Blastp revealed homology sequences for the putative ORF7 of the HCQD-2020 strain.

### 3.3. Phylogenetic Analysis Suggesting That HCQD-2020 Strain Might Be a Novel Species Belonging to the Alphacoronavirus Genus

**Figure 2:**
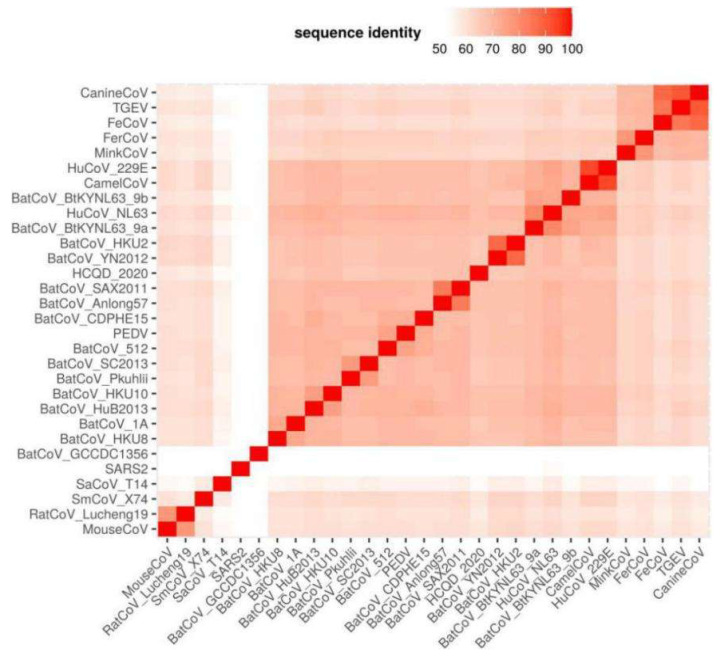
Heatmaps represented whole-genome comparisons between HCQD-2020 and other known species of *Alphacoronavirus* and *Betacoronavirus*. The color scale represents the sequence identity in percentage of each sequence pair. It is clear that the similarities between the HCQD-2020 strain and other strains were very low.

**Figure 3:**
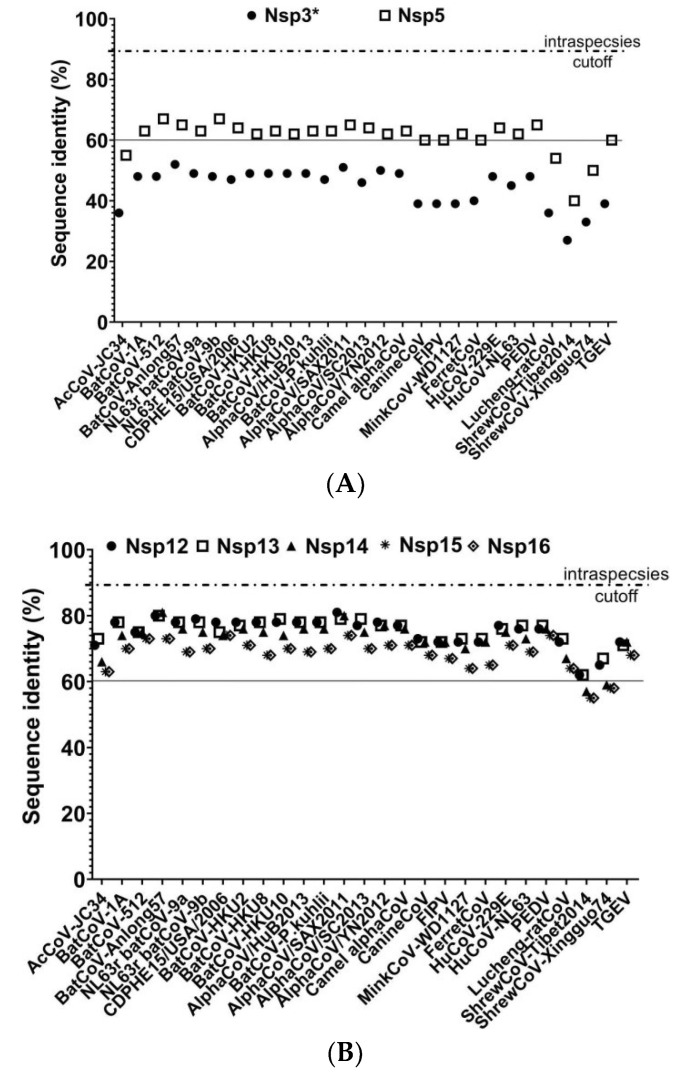
Amino acid sequence comparisons of conserved domains in replicated polyproteins between the HCQD-2020 strain and other alphacoronaviruses. The conserved regions of Nsp3 and Nsp5 in ORF1A (**A**) ad Nsp12–Nsp16 in ORF1b (**B**) were compared between HCQD-2020 strains and other reference strains.* For Nsp3: Only papain-like protease and ADP-ribose binding domains were applied for comparison. The solid lines are used to highlight the low similarity of Nsp3 and Nsp5 compared with the remaining. Nsps.

**Figure 4:**
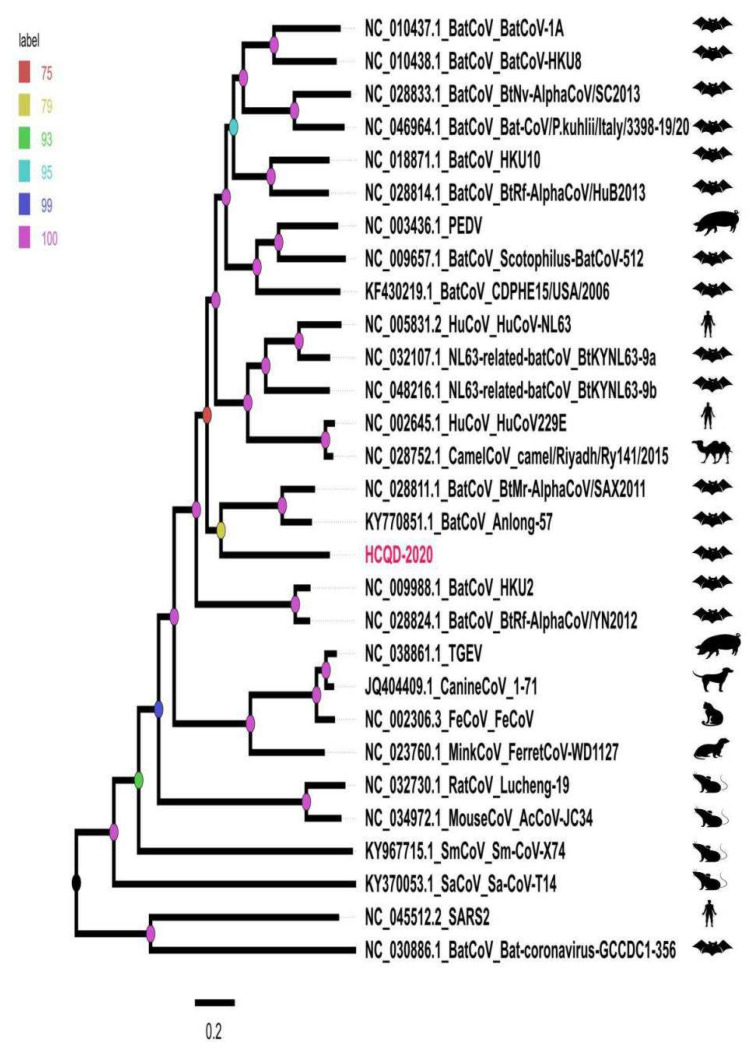
Phylogenetic tree representing the relationship among species and their hosts in the *Alphacorona* genus constructed from the whole-genome sequence. SARS-CoV2 and GCCDC1-356 belonging to *Betacoronavirus* were used as out group. The present strain is highlighted in red. The color of label represented the approximate bootstrap value in each node.

A whole-genome comparison indicated that our strain was mostly related to BatCoV Anlong57 (KY770851) and SAX2011 (NC_028811) with percentage identity values of 56.1% and 55.7%, respectively (Figure 2). Additionally, an amino acid comparison of seven highly conserved regions in replicate proteins of HCQD-2020 with other members of *Alphacoronavirus* indicated that the most similar regions of HCQD-2020 with other known species were in Nsp12, Nsp13, and Nsp14 (Figure 3B) atapproximately 80%, which is far below the cutoff value of a new species at 90% amino acid identity according to the International Committee on Taxonomy of Viruses (ICTV). In other regions, the amino acid sequence’s similarity was under 75% (Figure 3A,B). More specifically, Nsp3 was the most distantly related between HCQD-2020 and other strains, with amino acid identity ranking from 27 to 52%; followed by Nsp5, with a rank of 40–67% (Figure 3A). On the other hand, conserved regions located in ORF1b were more conserved between the present strain and others, with differences of about 19–45% (Figure 3B). Phylogeny based on the whole-genome sequence also indicated that our strain is distantly related to other known species belonging to *Alphacoronavirus* (Figure 4, Figure S2). This result suggests that HCQD-2020 might be a new species belonging to *Alphacoronavirus.*

**Figure 5:**
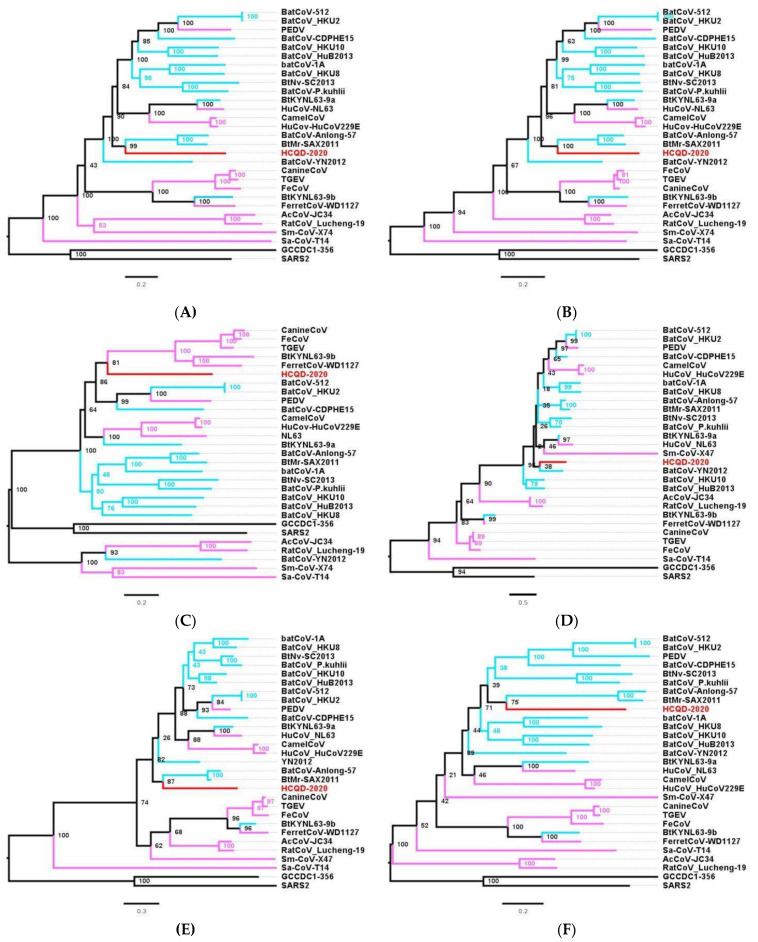
Phylogenetic analysis of the novel coronavirus based on the main nonstructural and structural protein-encoding genes. Phylogenetic tree construction based on the ORF1a (**A**), ORF1b (**B**), S (**C**), E (**D**), M (**E**), and N (**F**) genes, respectively. The present strain is highlighted in red. The coronavirus species originating from bats are presented in light blue while the nonbat mammalian host coronaviruses are labeled in pink. The scale bar indicates the number of substitutions per site.

Further classifications were conducted based on the topology of phylogeny constructed on the basis of two main nonstructural protein-encoding genes, ORF1a and ORF1b, and four main structural protein-encoding genes. Except for the highly similar topological trees based on ORF1a and ORF1b (Figure 5A,B), the remaining phylogenetic trees revealed the change in the position of the HCQD-2020 strain within the *Alphacoronavirus* genus (Figure 5C–F). Even so, this strain was placed on a separate branch from the other known species of *Alphacoronavirus*, further supporting that HCQD-2020 is likely a novel species.

### 3.4. In Silico Cross-Species Infectious Ability Examination

**Figure 6:**
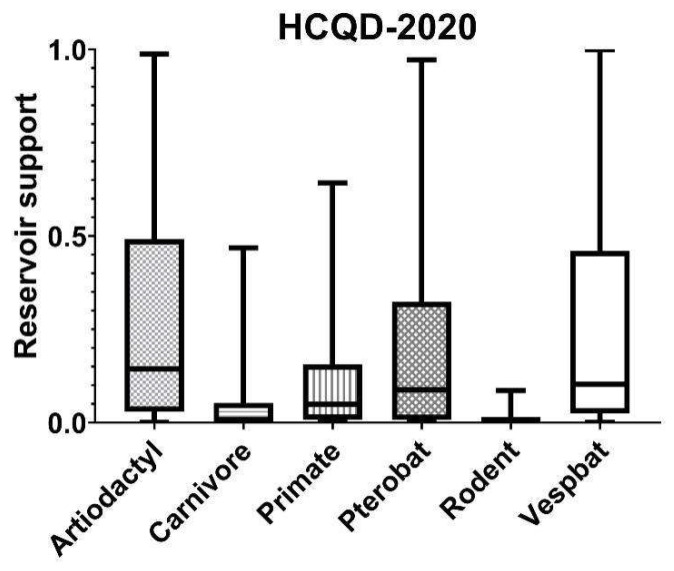
Potential host prediction of HCQD-2020 strain cross-infection in other orders of mammalians using viral host predictor tools. The boxes represent values between 25th and 75th percentiles of the probability scores while the whiskers show the min and max probability scores.

Bat coronaviruses are mostly significant due to their risk of zoonotic diseases. In this study, we applied an in silicoanalysis to predict the potential infection of this virus in another host. The results indicated that, in addition to its natural reservoir of microbats (*Vespertilioniformes*), this strain can also infect other hosts belonging to the order Artiodactyla (Figure 6) with equal probability. In detail, the q1, median, and q3 of the probability scores of the Artiodactyla host group were 0.03, 0.13, and 0.43, respectively, while the corresponding values for the Vespbat host group were 0.02, 0.1, and 0.44. These results indicate the risk of cross-species infection of the HCQD-2020 strain.

## 4. Discussion

The bat is considered to be a source of several viral pathogens transmissible to humans and livestock. The first evidence of rabies virus transmission from bats was reported in 1921 [43]. After that, an increasing number of human and animal viral diseases such as the Hendra virus [44], Nipah virus [45], and pteropine orthoreovirus [43] were detected in bats. Since the development of sequencing technology, as well as the emergence of the deadly pathogen SARS-CoV, studies related to the diversity of bat virome, including members with risks of zoonotic infection belonging to *Coronaviridae*, *Paramyxoviridae*, *Reoviridae*, *Rhabdoviridae*, and *Filoviridae* [19], have been elucidated. Climate change and human activities result in close contact between wild animals and humans, consequentially increasing the risk of host transmission of viruses. In brief, serological evidence revealed the multi-infection of SARS-related coronavirus from bats to humans [46,47]. Therefore, active vigilance against bat-borne viruses with added attention to the coronavirus is essential in the prevention of other widespread zoonotic diseases.

In this study, several species belonging to genera *Eptesicus, Myotis,* and *Pipistrellus* have been investigated for the presence of coronavirus. These species share their habitat niches with other wild and/or livestock animals, thereby increasing the risk of cross-contamination to humans involving any of the viruses they carry. Of these samples, a distantly genetically related viral isolate belonging to *Alphacoronavirus* was detected in *E. serotinus*. This result further contributed to the genetic diversity of bat coronavirus in general and *Alphacoronavirus* in particular.

It is generally accepted that the *Alphacoronavirus* genus is extremely diverse. To date, 19 different species belonging to 14 sub-genera of *Alphacoronavirus* have been officially accepted by ICTV. In this study, a whole-genome comparison indicated that the HCQD-2020 strain was distantly related to other known species of *Alphacoronavirus* (Figure 2). Genome-based and functionalgene-based phylogeny constructions also indicated that this strain formed a separate branch in phylogenetic trees (Figure 4 and Figure 5). Recent metagenomic studies of bat virome revealed several potential novel species within *Alphacoronavirus* detected in bats around the world [27,48,49,50]. This result, along with other up-to-date studies, once again supported the genetic heterogeneity of this genus.

All members of this genus have similar genomic organization, containing ORF1ab–S–ORF3–E–M–N [42]. Furthermore, additional ORFs located downstream of the nucleocapsid–encoding gene were also observed in many species of this genus such as TGEV, BatCoV-HKU2, BatCoV-512, and Shrew coronavirus [12]. In addition to the common ORFs found in other *Alphacoronavirus*’s members, a putative ORF7 was found at the 3′ terminator of HCQD-2020′s genome (Figure 1). Its sequence at the amino acid level was not homologous with any of the known protein sequences. It should also be noted that this putative ORF was likely the most distinct ORF of the currently known alphacoronavirus [12].

*Alphacoronavirus* contains several harmful viruses such as TGEV, PEDV, and PEAV that cause serious economic losses in pig production [4]. The last two species were considered to originate from bats [51,52]. Evidence of the host jumping of coronavirus from bat to other species belonging to even-toed ungulate animals was characterized in the case of PEAV, which shares high nucleotide identity (approximately 95% sequence similarity) with bat-HKU2 strains [53]. In this study, an in silico analysis indicated that HCQD-2020, a distantly related species belonging to *Alphacoronavirus*, can infect another host, especially those in the order Artiodactyla, which include some species such as camels and pigs (Figure 6). Camels were previously determined as the intermediate hosts of the MERS virus [54,55]. Focusing on the genus *Alphacoronavirus*, strains that were closely related to the human alphacorornavirus E229 were detected in domestic camels [56]. Recently, a novel alphacoronavirus belonging to the species *Alphacoronavirus I* that is usually found in pigs, dogs, and cats was detected in children with pneumonia in Malaysia [57]. Therefore, it is important to investigate potential hosts besides bats for newly detected coronaviruses.

## 5. Conclusions

In summary, this study reported and described the nearly complete genome of an *Alphacoronavirus* species originating from bats. Based on the low sequence identity, the presence of a putative ORF7 with no homology to any known genes in Genbank, and distant relation to other representative species of *Alphacoronavirus*, the HCQD-2020 strain was proposed as a novel strain of this genus. In silicoanalyses suggested that this newly identified strain of coronavirus could infect other hosts, not limited to bats. Future studies should focus on understanding the diversity of *coronavirus* and the probability of host jumping.

## Figures and Tables

**Table 1:** ORFs and TRS locations of novel coronavirus strain HCQD-2020.

ORFs	Length (nt/aa)	TRS Location	TRS Sequence(s) (Distance to ATG) ^1^
ORF1ab	20,477/6824	36	CCCCTC**AACTAAAC**GAA(215)ATG
S	4035/1344	20,731	GTTTC**AACCAAAT**GAAAAA
ORF3	675/224	24,723	AGTCG**AACTrAAAC**TCA(34)ATG
E	246/81	25,342	TATTG**AACTAAGT**GAC(61)ATG
M	678/225	25,658	TGTCT**AACTAAAT**CAA(1)ATG
N	1155/384	26,530	TAATC**AATTAAAC**AAA(4)ATG
ORF7	837/278	27,514	ACTC**AACTAAAC**ATG

^1^ The TRS sequences were highlighted as bold. The number indicated the distance in nucleotide from TRS to start codon. Initial codons are underlined.

**Table 2:** Secondary structural genomic elements of novel alphacoronavirus strain HCQD-2020.

RNA Structural Elements	Position	Rfam	Note
5′ UTR	1–289	RF03116	
-1 frameshift element	12,762–12,768		Conservative heptamer TTTAAAC
Frameshifting stimulation	12,770–12,848	RF00507	
3′ UTR	28,408–28,753	RF03121	

**Table 3:** Putative nonstructural proteins and the cleavage sites of polyproteins 1a and 1ab of the HCQD-2020 strain.

Nsp	First–Last Amino Acid Residues ^1^	Protein Size	Cleavage Sequence	Putative Functional Domains
Nsp1	M1-G282	282	GNVEAG|DVVFTS	Unknown function, PFAM: PF19211
Nsp2	D283-G890	608	FKRGGG|VTFGGD	Unknown function, PFAM: PF19212
Nsp3	V891–G2580	1690	IVQKSG|SGPQFP	Papain-like protease, PFAM: PF08715
Nsp4	S2581–Q3058	478	SSLQ|AGLR	Membrane spanning domain, PFAM: PF16348
Nsp5	A3059-Q3360	302	VTLQ|GGRK	3C-like protease, PFAM: PF05409
Nsp6	G3361–Q36439	279	SSVQ|SKLT	Membrane spanning protein, PFAM: PF19213
Nsp7	S3640–Q3722	83	AMLQ|SIAS	RNA replicate protein complex, PFAM: PF08716
Nsp8	S3723–Q3917	195	VKLQ|NNEV	Transferase activity, PFAM: PF08717
Nsp9	N3918–Q4026	109	IRLQ|AGKQ	Single strain RNA binding protein, PFAM: PF08710
Nsp10	A4027–Q4161	135	ANVQ|SFDQ	Nucleic-binding protein, PFAM: PF09401
Nsp11	A4162-D4178	17	-	Short peptide in C-terminate of ORF1a
Nsp12	A4162-Q5062	901	TVLQ|ASGM	RNA-depend RNA polymerase, PFAM: PF06478
Nsp13	A5063–Q5659	597	TDLQ|ATEG	Helicase, Interpro: IRP027351
Nsp14	A5660–Q6178	519	TKIQ|GLEN	Exoribonuclease and Guanine-N7 methyltransferase, Interpro: IPR009466
Nsp15	G6179-Q6526	347	PQLQ|SAEW	EndoU-like endoribonuclease, PFAM: PF19215
Nsp16	S6526–K6825	300	-	O-methyltransferase, PFAM: PF06460

^1^ Number indicated the position of amino acid in the nonstructural protein 1a or 1ab.

## Data Availability

Data are contained within article and supplementary material.

## Supplementary Materials

The following are available online at https://www.mdpi.com/article/10.3390/v13102041/s1, Figure S1: Initial classification of HCQD-2020 strain within subfamily Coronavirinae using the conserved region of Rdrp encoding fragment, Figure S2: Phylogenetic tree construction based on whole genome sequence analysis, Table S1: Information of bat samples collected in this study, Table S2: List of primers using in this study.

## References

[1] Cui J., Li F., Shi Z.L. (2019). Origin and evolution of pathogenic coronaviruses. Nat. Rev. Microbiol..

[2] Ghai R.R., Carpenter A., Liew A.Y., Martin K.B., Herring M.K., Gerber S.I., Hall A.J., Sleeman J.M., VonDobschuetz S., Behravesh C.B. (2021). Animal reservoirs and hosts for emerging alphacoronaviruses and betacoronaviruses. Emerg. Infect. Dis..

[3] Zhang M.X., Xie Z.X., Xie L.J., Deng X.W., Xie Z.Q., Luo S.S., Liu J.B., Pang Y.S., Khan M.I. (2015). Simultaneous detection of eight swine reproductive and respiratory pathogens using a novel gexp analyser-based multiplex pcr assay. J. Virol. Methods.

[4] Vlasova A.N., Wang Q., Jung K., Langel S.N., Malik Y.S., Saif L.J. (2020). Porcine coronaviruses. Emerg. Transbound. Anim. Viruses.

[5] Woo P.C., Lau S.K., Huang Y., Yuen K.-Y. (2009). Coronavirus Diversity, Phylogeny and Interspecies Jumping. Exp. Biol. Med..

[6] Anthony S.J., Johnson C.K., Greig D.J., Kramer S., Che X., Wells H., Hicks A.L., Joly D.O., Wolfe N.D., Daszak P. (2017). Global patterns in coronavirus diversity. Virus Evol..

[7] Monchatre-Leroy E., Boué F., Boucher J.M., Renault C., Moutou F., Ar Gouilh M., Umhang G. (2017). Identification of alpha and beta coronavirus in wildlife species in france: Bats, rodents, rabbits, and hedgehogs. Viruses.

[8] Wang W., Lin X.D., Zhang H.L., Wang M.R., Guan X.Q., Holmes E.C., Zhang Y.Z. (2020). Extensive genetic diversity and host range of rodent-borne coronaviruses. Virus Evol..

[9] Smith C.S., de Jong C.E., Meers J., Henning J., Wang L.F., Field H.E. (2016). Coronavirus infection and diversity in bats in the australasian region. EcoHealth.

[10] Wang W., Lin X.-D., Guo W.-P., Zhou R.-H., Wang M.-R., Wang C.-Q., Ge S., Mei S.-H., Li M.-H., Shi M. (2015). Discovery, diversity and evolution of novel coronaviruses sampled from rodents in china. Virology.

[11] Lin X.D., Wang W., Hao Z.Y., Wang Z.X., Guo W.P., Guan X.Q., Wang M.R., Wang H.W., Zhou R.H., Li M.H. (2017). Extensive diversity of coronaviruses in bats from china. Virology.

[12] Wang W., Lin X.D., Liao Y., Guan X.Q., Guo W.P., Xing J.G., Holmes E.C., Zhang Y.Z. (2017). Discovery of a highly divergent coronavirus in the asian house shrew from china illuminates the origin of the alphacoronaviruses. J. Virol..

[13] Woo P.C., Lau S.K., Li K.S., Poon R.W., Wong B.H., Tsoi H.W., Yip B.C., Huang Y., Chan K.H., Yuen K.Y. (2006). Molecular diversity of coronaviruses in bats. Virology.

[14] So R.T.Y., Chu D.K.W., Miguel E., Perera R., Oladipo J.O., Fassi-Fihri O., Aylet G., Ko R.L.W., Zhou Z., Cheng M.S. (2019). Diversity of Dromedary Camel Coronavirus HKU23 in African Camels Revealed Multiple Recombination Events among Closely Related Betacoronaviruses of the Subgenus Embecovirus. J. Virol..

[15] Borucki M.K., Allen J.E., Chen-Harris H., Zemla A., Vanier G., Mabery S., Torres C., Hullinger P., Slezak T. (2013). The Role of Viral Population Diversity in Adaptation of Bovine Coronavirus to New Host Environments. PLoS ONE.

[16] Wu Z., Yang L., Ren X., He G., Zhang J., Yang J., Qian Z., Dong J., Sun L., Zhu Y. (2016). Deciphering the bat virome catalog to better understand the ecological diversity of bat viruses and the bat origin of emerging infectious diseases. ISME J..

[17] Boni M.F., Lemey P., Jiang X., Lam T.T., Perry B.W., Castoe T.A., Rambaut A., Robertson D.L. (2020). Evolutionary origins of the SARS-CoV-2 sarbecovirus lineage responsible for the COVID-19 pandemic. Nat. Microbiol..

[18] Woo P.C.Y., Lau S.K.P., Lam C.S.F., Lau C.C.Y., Tsang A.K.L., Lau J.H.N., Bai R., Teng J.L.L., Tsang C.C.C., Wang M. (2012). Discovery of Seven Novel Mammalian and Avian Coronaviruses in the Genus Deltacoronavirus Supports Bat Coronaviruses as the Gene Source of Alphacoronavirus and Betacoronavirus and Avian Coronaviruses as the Gene Source of Gammacoronavirus and Deltacoronavirus. J. Virol..

[19] Letko M., Seifert S.N., Olival K.J., Plowright R.K., Munster V.J. (2020). Bat-borne virus diversity, spillover and emergence. Nat. Rev. Microbiol..

[20] Lo V.T., Yoon S.W., Noh J.Y., Kim Y., Choi Y.G., Jeong D.G., Kim H.K. (2020). Long-term surveillance of bat coronaviruses in korea: Diversity and distribution pattern. Transbound Emerg. Dis..

[21] Maganga G.D., Pinto A., Mombo I.M., Madjitobaye M., Mbeang Beyeme A.M., Boundenga L., Ar Gouilh M., N’Dilimabaka N., Drexler J.F., Drosten C. (2020). Genetic diversity and ecology of coronaviruses hosted by cave-dwelling bats in gabon. Sci. Rep..

[22] Góes L.G.B., Campos A.C.A., Carvalho C., Ambar G., Queiroz L.H., Cruz-Neto A.P., Munir M., Durigon E.L. (2016). Genetic diversity of bats coronaviruses in the atlantic forest hotspot biome, brazil. Infect. Genet. Evol..

[23] Tang X.C., Zhang J.X., Zhang S.Y., Wang P., Fan X.H., Li L.F., Li G., Dong B.Q., Liu W., Cheung C.L. (2006). Prevalence and Genetic Diversity of Coronaviruses in Bats from China. J. Virol..

[24] Woo P.C., Lau S.K., Yuen K.Y. (2006). Infectious diseases emerging from chinese wet-markets: Zoonotic origins of severe respiratory viral infections. Curr. Opin. Virol..

[25] Gloza-Rausch F., Ipsen A., Seebens A., Gottsche M., Panning M., Drexler J.F., Petersen N., Annan A., Grywna K., Muller M. (2008). Detection and prevalence patterns of group I coronaviruses in bats, northern Germany. Emerg. Infect. Dis..

[26] Lau S.K., Li K.S., Tsang A.K., Shek C.T., Wang M., Choi G.K., Guo R., Wong B.H., Poon R.W., Lam C.S. (2012). Recent transmission of a novel alphacoronavirus, bat coronavirus hku10, from leschenault’s rousettes to pomona leaf-nosed bats: First evidence of interspecies transmission of coronavirus between bats of different suborders. J. Virol..

[27] Zhou H., Ji J., Chen X., Bi Y., Li J., Wang Q., Hu T., Song H., Zhao R., Chen Y. (2021). Identification of novel bat coronaviruses sheds light on the evolutionary origins of sars-cov-2 and related viruses. Cell.

[28] Lee S., Jo S.D., Son K., An I., Jeong J., Wang S.J., Kim Y., Jheong W., Oem J.K. (2018). Genetic characteristics of coronaviruses from korean bats in 2016. Microb. Ecol..

[29] Poon L.L., Chu D.K., Chan K.H., Wong O.K., Ellis T.M., Leung Y.H., Lau S.K., Woo P.C., Suen K.Y., Yuen K.Y. (2005). Identification of a novel coronavirus in bats. J. Virol..

[30] Bankevich A., Nurk S., Antipov D., Gurevich A.A., Dvorkin M., Kulikov A.S., Lesin V.M., Nikolenko S.I., Pham S., Prjibelski A.D. (2012). Spades: A new genome assembly algorithm and its applications to single-cell sequencing. J. Comput. Biol..

[31] Scotto–Lavino E., Du G., Frohman M.A. (2006). 5′ end cdna amplification using classic race. Nat. Protoc..

[32] Chen L.-L., Ou H.-Y., Zhang R., Zhang C.-T. (2003). Zcurve_cov: A new system to recognize protein coding genes in coronavirus genomes, and its applications in analyzing sars-cov genomes. Biochem. Biophys. Res. Commun..

[33] Mitchell A.L., Attwood T.K., Babbitt P.C., Blum M., Bork P., Bridge A., Brown S.D., Chang H.Y., El-Gebali S., Fraser M.I. (2019). Interpro in 2019: Improving coverage, classification and access to protein sequence annotations. Nucleic Acids Res..

[34] Kalvari I., Nawrocki E.P., Ontiveros-Palacios N., Argasinska J., Lamkiewicz K., Marz M., Griffiths-Jones S., Toffano-Nioche C., Gautheret D., Weinberg Z. (2021). Rfam 14: Expanded coverage of metagenomic, viral and microrna families. Nucleic Acids Res..

[35] Graham R.L., Baric R.S. (2010). Recombination, Reservoirs, and the Modular Spike: Mechanisms of Coronavirus Cross-Species Transmission. J. Virol..

[36] Su S., Wong G., Shi W., Liu J., Lai A.C.K., Zhou J., Liu W., Bi Y., Gao G.F. (2016). Epidemiology, Genetic Recombination, and Pathogenesis of Coronaviruses. Trends Microbiol..

[37] Katoh K., Standley D.M. (2013). MAFFT multiple sequence alignment software version 7: Improvements in performance and usability. Mol. Biol. Evol..

[38] Minh B.Q., Schmidt H.A., Chernomor O., Schrempf D., Woodhams M.D., Von Haeseler A., Lanfear R. (2020). IQ-TREE 2: New models and efficient methods for phylogenetic inference in the genomic era. Mol. Biol. Evol..

[39] Kalyaanamoorthy S., Minh B.Q., Wong T.K.F., von Haeseler A., Jermiin L.S. (2017). ModelFinder: Fast model selection for accurate phylogenetic estimates. Nat. Methods.

[40] Hoang D.T., Chernomor O., von Haeseler A., Minh B.Q., Vinh L.S. (2018). UFBoot2: Improving the ultrafast bootstrap approximation. Mol. Biol. Evol..

[41] Babayan S.A., Orton R.J., Streicker D.G. (2018). Predicting reservoir hosts and arthropod vectors from evolutionary signatures in rna virus genomes. Science.

[42] King A.M.Q., Adams M.J., Carstens E.B., Lefkowitz E.J. (2012). Family—Coronaviridae. Virus Taxonomy.

[43] Tan C.W., Yang X., Anderson D.E., Wang L.F. (2021). Bat virome research: The past, the present and the future. Curr. Opin. Virol..

[44] Halpin K., Young P.L., Field H.E., Mackenzie J.S. (2000). Isolation of hendra virus from pteropid bats: A natural reservoir of hendra virus. J. Gen. Virol..

[45] Yob J.M., Field H., Rashdi A.M., Morrissy C., van der Heide B., Rota P., bin Adzhar A., White J., Daniels P., Jamaluddin A. (2001). Nipah virus infection in bats (order Chiroptera) in peninsular Malaysia. Emerg. Infect. Dis..

[46] Li H., Mendelsohn E., Zong C., Zhang W., Hagan E., Wang N., Li S., Yan H., Huang H., Zhu G. (2019). Human-animal interactions and bat coronavirus spillover potential among rural residents in Southern China. Biosaf Health.

[47] Wang N., Li S.Y., Yang X.L., Huang H.M., Zhang Y.J., Guo H., Luo C.M., Miller M., Zhu G., Chmura A.A. (2018). Serological Evidence of Bat SARS-Related Coronavirus Infection in Humans, China. Virol. Sin..

[48] Bergner L.M., Orton R.J., Streicker D.G. (2020). Complete Genome Sequence of an Alphacoronavirus from Common Vampire Bats in Peru. Microbiol. Resour. Announc..

[49] Lazov C.M., Belsham G.J., Botner A., Rasmussen T.B. (2021). Full-Genome Sequences of Alphacoronaviruses and Astroviruses from Myotis and Pipistrelle Bats in Denmark. Viruses.

[50] Prada D., Boyd V., Baker M.L., O’Dea M., Jackson B. (2019). Viral Diversity of Microbats within the South West Botanical Province of Western Australia. Viruses Basel.

[51] Fu X., Fang B., Liu Y., Cai M., Jun J., Ma J., Bu D., Wang L., Zhou P., Wang H. (2018). Newly emerged porcine enteric alphacoronavirus in southern China: Identification, origin and evolutionary history analysis. Infect. Genet. Evol..

[52] Huang Y.W., Dickerman A.W., Pineyro P., Li L., Fang L., Kiehne R., Opriessnig T., Meng X.J. (2013). Origin, evolution, and genotyping of emergent porcine epidemic diarrhea virus strains in the united states. mBio.

[53] Gong L., Li J., Zhou Q., Xu Z., Chen L., Zhang Y., Xue C., Wen Z., Cao Y. (2017). A new bat-HKU2–like coronavirus in swine, China, 2017. Emerg. Infect. Dis..

[54] Alagaili A.N., Briese T., Mishra N., Kapoor V., Sameroff S.C., Burbelo P.D., de Wit E., Munster V.J., Hensley L.E., Zalmout I.S. (2014). Middle East respiratory syndrome coronavirus infection in dromedary camels in Saudi Arabia. mBio.

[55] Alshukairi A.N., Zheng J., Zhao J., Nehdi A., Baharoon S.A., Layqah L., Bokhari A., Al Johani S.M., Samman N., Boudjelal M. (2018). High Prevalence of MERS-CoV Infection in Camel Workers in Saudi Arabia. mBio.

[56] Sabir J.S.M., Lam T.T.-Y., Ahmed M.M.M., Li L., Shen Y., Abo-Aba S.E.M., Qureshi M.I., Abu-Zeid M., Zhang Y., Khiyami M.A. (2016). Co-circulation of three camel coronavirus species and recombination of mers-covs in saudi arabia. Science.

[57] Vlasova A.N., Diaz A., Damtie D., Xiu L., Toh T.H., Lee J.S., Saif L.J., Gray G.C. (2021). Novel Canine Coronavirus Isolated from a Hospitalized Pneumonia Patient, East Malaysia. Clin. Infect. Dis..

