# Supplementary material for: Genomic Characterization of a Novel Alphacoronavirus Isolated from Bats, Korea, 2020
Source: Viruses. 2021 Oct 11;13(10):2041. doi: 10.3390/v13102041 (PMC8540747; doi:10.3390/v13102041)
Supplement: Supplementary file 1 [file viruses-13-02041-s001.zip › viruses-1334791-supplementary-data.pdf]

Supplement data. Reference coronavirus using in this study.

No	Strain	Date collection	Host	Accession number
1	Rousettus bat coronavirus	2014	Rousettus leschenaulti	NC_030886.1
2	SARS-CoV-2	2019	human	NC_045512.2
3	Wencheng Sm shrew coronavirus isolate Xingguo-101	2015	Suncus murinus	NC_035191.1
4	Human coronavirus 229E strain HCoV_229E/Seattle/USA/SC677/2016	2016	Homo sapiens	KY369909.2
5	Rhinolophus affinis bat coronavirus HKU2-related isolate 160660	2018	Rhinolophus affinis	MN611522.1
6	BtRf-AlphaCoV/YN2012	2012	Rhinolophus ferrumequinum	NC_028824.1
7	Swine acute diarrhea syndrome coronavirus isolate SADS-CoV/GDWT-P83	2018	Sus scrofa	MK994937.1
8	Camel alphacoronavirus Camel229E isolate Camel229E-CoV/KCSP1/KEN/2015	2015	Camelus dromedarius	KU291449.1
9	Bat coronavirus HKU2 strain HKU2/HK/46/2006			EF203065.1
10	Bat coronavirus HKU2			NC_009988.1
11	Human coronavirus 229E strain 229E/human/USA/933-50/1993	1993	Homo sapiens	KF514430.1
12	Swine acute diarrhea syndrome coronavirus isolate SADS-CoV-CH-FJWT-2018	2018	Sus scrofa	MH615810.1
13	Alphacoronavirus sp. strain VZ_AlphaCoV_16715_84	2014	Scotophilus kuhlii	MH687952.1
14	Human coronavirus 229E strain HCoV_229E/Seattle/USA/SC9773/2016	2016	Homo sapiens	KY369914.1
15	Mutant Swine acute diarrhea syndrome coronavirus strain icSADS-tRFP		Sus scrofa	MT039232.1
16	Human coronavirus 229E strain 229E/Haiti-1/2016	2016	Homo sapiens	MF542265.1
17	Alphacoronavirus sp. strain VZ_AlphaCoV_17819_22	2014	Scotophilus kuhlii	MH687960.1
18	Human coronavirus 229E isolate HCoV-229E/USA/UAMS_DID_0108/2017	2017	Homo sapiens	MT438700.1
19	Human coronavirus 229E			NC_002645.1
20	Alpaca respiratory coronavirus isolate CA08-1/2008	2008	Vicugna pacos	JQ410000.1
21	Camel alphacoronavirus isolate camel/Jeddah/Jd63/2014	2014	Camelus	KT368894.1
22	Alphacoronavirus sp. isolate WA3301	2018		MK472069.1
23	Alphacoronavirus sp. isolate WA2028	2018		MK472068.1
24	Porcine epidemic diarrhea virus strain USA/OK10240-8/2017	2017	Sus scrofa	MG334555.1
25	Porcine epidemic diarrhea virus genomic RNA, strain: MYG-1/JPN/2014	2014	Sus scrofa domesticus	LC063838.1
26	Human coronavirus NL63 strain HCoV_NL63/Seattle/USA/SC0179/2018	2018	Homo sapiens	MN306018.1
27	Human coronavirus NL63 strain HCoV_NL63/Seattle/USA/SC2940/2015	2015	Homo sapiens	KY983586.1
28	Human coronavirus NL63 strain ChinaGD04	2018	Homo sapiens	MK334047.1

29	Porcine epidemic diarrhea virus strain 85-7-mutant2	2015		KX839248.1
30	Alphacoronavirus sp. isolate WAAlc1	2018		MK472071.1
31	Human coronavirus NL63 strain HCoV_NL63/Seattle/USA/SC0768/2019	2019	Homo sapiens	MN306040.1
32	Human coronavirus NL63 strain ChinaGD05	2018	Homo sapiens	MK334045.1
33	Human coronavirus NL63 isolate Amsterdam 057			DQ445911.1
34	PRCV ISU-1		Sus scrofa	DQ811787.1
35	Human Coronavirus NL63			NC_005831.2
36	Human group 1 coronavirus associated with pneumonia			AY518894.1
37	Lucheng Rn rat coronavirus isolate Lijiang-170	2015	Apodemus chevrieri	MT820625.1
38	BtMs-AlphaCoV/GS2013	2013	Myotis sp.	KJ473810.1
39	BtRf-AlphaCoV/HuB2013	2013	Rhinolophus ferrumequinum	NC_028814.1
40	Rousettus aegyptiacus bat coronavirus 229E-related isolate 5425	2018	Rousettus aegyptiacus	MN611517.1
41	Tylonycteris bat coronavirus HKU33 strain GZ151867	2015	Tylonycteris robustula	MK720944.1
42	229E-related bat coronavirus strain BtKY229E-8	2010	Macronycteris vittata	KY073748.1
43	Porcine epidemic diarrhea virus strain PEDV/MEX/GTO/02/2016	2016	Sus scrofa	MH004412.1
44	Porcine epidemic diarrhea virus strain 17GXCZ-1ORF3d	2017	Sus scrofa	MT547179.1
45	Coronavirus AcCoV-JC34	2011	Apodemus chevrieri	NC_034972.1
46	Alphacoronavirus sp. strain VZ_AlphaCoV_20745_8	2015	Scotophilus kuhlii	MH687967.1
47	Alphacoronavirus sp. strain VZ_AlphaCoV_17819_50	2014	Scotophilus kuhlii	MH687962.1
48	Porcine epidemic diarrhea virus isolate 4-2	2013	Sus scrofa	MW165329.1
49	Porcine respiratory coronavirus strain OH7269	2014	Sus scrofa	KR270796.1
50	Porcine respiratory coronavirus strain PRCV/USA/Minnesota-46140/2016	2016		KY406735.1
51	BtNv-AlphaCoV/SC2013	2013	Nyctalus velutinus	NC_028833.1
52	Human coronavirus NL63 isolate Kilifi_HH_5402_20-May-2010	2010	Homo sapiens	MG428704.1
53	229E-related bat coronavirus strain BtKY229E-1	2009	Hipposideros	KY073747.1
54	Porcine epidemic diarrhea virus strain 85-7-mutant4	2015		KX839250.1
55	Porcine epidemic diarrhea virus isolate JSLS-1/2015	2015	Sus scrofa	KX534205.1
56	Alphacoronavirus Bat-CoV/P.kuhlii/Italy/206645-41/2011	2011	Pipistrellus kuhlii	MH938448.1
57	TGEV Miller M60	1987	Sus scrofa	DQ811786.2
58	Scotophilus kuhlii bat coronavirus 512-related isolate HK140714	2018	Scotophilus kuhlii	MN611521.1
59	BtMr-AlphaCoV/SAX2011	2011	Myotis ricketti	NC_028811.1
60	Porcine epidemic diarrhea virus strain AH-	2018	Sus scrofa	MN315264.1

	2018-HF1			
61	Porcine epidemic diarrhea virus strain HUA-14PED96	2014	Sus scrofa	KT941120.1
62	Porcine epidemic diarrhea virus isolate CO/P14/IC	2013	Sus scrofa	KU558702.1
63	Porcine epidemic diarrhea virus strain shxx1902	2019	Sus scrofa	MT303066.1
64	Porcine epidemic diarrhea virus	2019	Sus scrofa	MT843280.1
65	Porcine epidemic diarrhea virus isolate KNU-141112-S DEL2/ORF3	2016	Sus scrofa	KY825241.1
66	Porcine epidemic diarrhea virus strain Italy/7239/2009	2009	Sus scrofa	KR061458.1
67	Ferret systemic coronavirus strain FSCV6	2015	Mustela putorius furo	KX512810.1
68	Porcine epidemic diarrhea virus strain CH/HNQX-3/14	2015	Sus scrofa	KR095279.1
69	Porcine epidemic diarrhea virus isolate KNU-1705	2017	Sus scrofa	MH052684.1
70	Porcine epidemic diarrhea virus isolate PEDV SH	2016	Sus scrofa	MK841494.1
71	Alphacoronavirus sp. strain VZ_AlphaCoV_16715_47_c1	2014	Scotophilus kuhlii	MH687941.1
72	Alphacoronavirus sp. isolate WA3607	2018		MK472070.1
73	Porcine epidemic diarrhea virus strain 85-7	2013	Sus scrofa	KX839246.1
74	Porcine epidemic diarrhea virus isolate KNU-1807	2018	Sus scrofa	MH243319.1
75	Porcine epidemic diarrhea virus isolate PEDV-Hjms	2015	Sus scrofa	KY007139.1
76	Porcine epidemic diarrhea virus strain HNAY2016		Sus scrofa	MT338518.1
77	Porcine epidemic diarrhea virus isolate CH/TP-3-1/2018	2018	Sus scrofa	MK140812.1
78	Porcine epidemic diarrhea virus strain HB2018	2018	Sus scrofa	MT166307.1
79	Porcine epidemic diarrhea virus isolate GDS09	2014	Sus scrofa	MH726408.1
80	Porcine epidemic diarrhea virus strain CH/SCMY/2018	2018	Sus scrofa	MH061343.1
81	Porcine epidemic diarrhea virus strain CH/S	1986	Sus scrofa	JN547228.1
82	Porcine epidemic diarrhea virus isolate KNU-1815	2018	Sus scrofa	MK032689.1
83	Porcine epidemic diarrhea virus strain USA/IL20697/2014 Passage 5	2013	Sus scrofa	KT860508.1
84	Porcine epidemic diarrhea virus strain virulent DR13	2009	Sus scrofa	JQ023161.1
85	Porcine epidemic diarrhea virus isolate KNU-1710	2017	Sus scrofa	MH052689.1
86	Bat coronavirus CDPHE15/USA/2006	2006	Myotis lucifugus	NC_022103.1
87	Porcine epidemic diarrhea virus strain CH/HNXX/2016		Sus scrofa	MT338517.1
88	Porcine epidemic diarrhea virus strain CH/SCZG/2017	2017	Sus scrofa	MH061337.1
89	Porcine epidemic diarrhea virus strain CH/SCLS/2018	2018	Sus scrofa	MH061341.1

90	Porcine epidemic diarrhea virus isolate CHSD2014	2014	Sus scrofa	KX791060.1
91	Porcine epidemic diarrhea virus clone VN/JFP1013_1/2013/Vinh An/Vietnam	2013	Sus scrofa	KJ960178.1
92	Porcine epidemic diarrhea virus isolate CH/TP-4-4/2018	2018	Sus scrofa	MK140814.1
93	Porcine epidemic diarrhea virus isolate SDSX16	2016	Sus scrofa	MH117940.1
94	Porcine epidemic diarrhea virus isolate KNU-1904	2019	Sus scrofa	MN971595.1
95	Porcine epidemic diarrhea virus isolate CT P10	2018	Sus scrofa	MN114121.1
96	Porcine epidemic diarrhea virus strain HeN170821	2017	Sus scrofa	MK862249.1
97	Porcine epidemic diarrhea virus isolate GDS07	2014	Sus scrofa	MH726370.1
98	Porcine epidemic diarrhea virus isolate GDS11	2014	Sus scrofa	MH726374.1
99	Porcine epidemic diarrhea virus isolate GDS27	2014	Sus scrofa	MH726380.1
100	Porcine epidemic diarrhea virus isolate GDS47	2016	Sus scrofa	MH726382.1
101	Porcine epidemic diarrhea virus isolate GDS50	2017	Sus scrofa	MH726383.1
102	Porcine epidemic diarrhea virus isolate GDS31	2015	Sus scrofa	MH726394.1
103	Porcine epidemic diarrhea virus isolate GDS49	2017	Sus scrofa	MH726406.1
104	Porcine epidemic diarrhea virus isolate CH-HB1-2018	2018	Sus scrofa	MK606368.1
105	Porcine epidemic diarrhea virus isolate KNU-1709	2017	Sus scrofa	MH052688.1
106	Porcine epidemic diarrhea virus isolate KUPE21	2001	Sus scrofa	MF737355.1
107	Porcine epidemic diarrhea virus isolate XM2-4	2016	Sus scrofa	KX812524.1
108	Porcine epidemic diarrhea virus strain KB2013-4	2013	Sus scrofa	KX580953.1
109	Porcine epidemic diarrhea virus strain PEDV-LY	2014	Sus scrofa	KM609210.1
110	Porcine epidemic diarrhea virus strain CH/FJZZ-9/2012	2012	Sus scrofa	KC140102.1
111	Porcine epidemic diarrhea virus strain CH/FJND-3/2011	2011	Sus scrofa	JQ282909.1
112	Porcine epidemic diarrhea virus strain LZC from China			EF185992.1
113	Porcine epidemic diarrhea virus isolate M3-SX2017	2017	Sus scrofa	MK644603.1
114	Porcine epidemic diarrhea virus strain 17GXCZ-1ORF3c	2017	Sus scrofa	MT547180.1
115	Porcine epidemic diarrhea virus isolate N7-GD2017	2017	Sus scrofa	MK644604.1
116	Porcine epidemic diarrhea virus strain B5-	2017	Sus scrofa	MF807952.1

	HB2017			
117	Porcine epidemic diarrhea virus strain XY2013	2013	Sus scrofa	KR818832.1
118	Porcine epidemic diarrhea virus strain PEDV-1C	2012	Sus scrofa	KM609203.1
119	Porcine epidemic diarrhea virus strain PEDV-8C		Sus scrofa	KM609205.1
120	Alphacoronavirus sp. strain VZ_AlphaCoV_16845_53	2014	Scotophilus kuhlii	MH687956.1
121	Porcine epidemic diarrhea virus strain SHXX1902	2019	Sus scrofa	MN841671.1
122	UNVERIFIED: Porcine epidemic diarrhea virus strain SX-WH	2018	Sus scrofa	MN594506.1
123	Porcine epidemic diarrhea virus isolate KNU-1601	2016	Sus scrofa	KY963963.1
124	Porcine epidemic diarrhea virus strain GD-XL-2019	2019	Sus scrofa	MN759311.1
125	Porcine epidemic diarrhea virus strain CH/SXYL/2016	2016	Sus scrofa	MF462814.1
126	Porcine epidemic diarrhea virus isolate CH/HBTS/2017	2017	Sus scrofa	MH581489.1
127	Porcine epidemic diarrhea virus strain GDgh			MG983755.1
128	Porcine epidemic diarrhea virus strain WHLL	2017	Sus scrofa	MN037494.1
129	Porcine epidemic diarrhea virus isolate CH/JXJA/2017	2017	Sus scrofa	MF375374.1
130	Swine enteric coronavirus strain Italy/213306/2009	2009	Sus scrofa	NC_028806.1
131	Alphacoronavirus sp. strain VZ_AlphaCoV_20745_17	2015	Scotophilus kuhlii	MH687965.1
132	Bat alphacoronavirus strain BtCoV/008_16/M.bra/FIN/2016	2016	Myotis brandtii	MN065811.1
133	Porcine epidemic diarrhea virus isolate CH/HNZZ47/2016	2016	Sus scrofa	KX981440.1
134	Alphacoronavirus Bat-CoV/P.kuhlii/Italy/3398-19/2015	2015	Pipistrellus kuhlii	NC_046964.1
135	Coronavirus BtSk-AlphaCoV/GX2018C	2017	Scotophilus kuhlii	MK211371.1
136	Alphacoronavirus Bat-CoV/P.kuhlii/Italy/206679-3/2010	2010	Pipistrellus kuhlii	MH938450.1
137	Ferret enteric coronavirus strain FECV1	2015	Mustela putorius furo	KX512809.1
138	Alphacoronavirus sp. strain VZ_AlphaCoV_16715_77	2014	Scotophilus kuhlii	MH687950.1
139	Alphacoronavirus sp. strain VZ_AlphaCoV_17819_4	2014	Scotophilus kuhlii	MH687961.1
140	Hipposideros pomona bat coronavirus CHB25 isolate CHB0025	2018	Hipposideros larvatus	MN611525.1
141	Alphacoronavirus sp. isolate WA1087	2018		MK472067.1
142	Alphacoronavirus sp. strain VZ_AlphaCoV_16715_61	2014	Scotophilus kuhlii	MH687946.1
143	Alphacoronavirus sp. strain VZ_AlphaCoV_16845_64	2014	Scotophilus kuhlii	MH687957.1
144	Myotis lucifugus coronavirus	2010	Myotis lucifugus	KY799179.1
145	Coronavirus BtSk-AlphaCoV/GX2018B	2017	Scotophilus kuhlii	MK211370.1

146	Alphacoronavirus sp. strain VZ_AlphaCoV_16715_76	2014	Scotophilus kuhlii	MH687949.1
147	Porcine epidemic diarrhea virus isolate GER/L03208/2019			LR812930.1
148	Scotophilus bat coronavirus 512			NC_009657.1
149	Alphacoronavirus sp. strain VZ_AlphaCoV_20745_10	2015	Scotophilus kuhlii	MH687964.1
150	Alphacoronavirus sp. strain VZ_AlphaCoV_16715_53	2014	Scotophilus kuhlii	MH687944.1
151	Alphacoronavirus sp. strain VZ_AlphaCoV_16845_47	2014	Scotophilus kuhlii	MH687955.1
152	Alphacoronavirus sp. strain VZ_AlphaCoV_16715_56	2014	Scotophilus kuhlii	MH687945.1
153	Alphacoronavirus sp. strain VZ_AlphaCoV_16715_32	2014	Scotophilus kuhlii	MH687937.1
154	Coronavirus BtSk-AlphaCoV/GX2018D	2017	Scotophilus kuhlii	MK211372.1
155	Alphacoronavirus sp. strain VZ_AlphaCoV_16715_5	2014	Scotophilus kuhlii	MH687943.1
156	Alphacoronavirus sp. strain VZ_AlphaCoV_16845_24	2014	Scotophilus kuhlii	MH687954.1
157	Alphacoronavirus sp. strain VZ_AlphaCoV_16715_23	2014	Scotophilus kuhlii	MH687934.1
158	Alphacoronavirus sp. strain VZ_AlphaCoV_16715_7	2014	Scotophilus kuhlii	MH687948.1
159	Coronavirus BtSk-AlphaCoV/GX2018A	2017	Scotophilus kuhlii	MK211369.1
160	Alphacoronavirus sp. strain VZ_AlphaCoV_16715_39_c2	2014	Scotophilus kuhlii	MH687939.1
161	BtMf-AlphaCoV/AH2011	2011	Miniopterus fuliginosus	KJ473795.1
162	BtMf-AlphaCoV/JX2012	2012	Miniopterus fuliginosus	KJ473796.1
163	Alphacoronavirus sp. strain VZ_AlphaCoV_16715_78	2014	Scotophilus kuhlii	MH687951.1
164	Porcine epidemic diarrhea virus strain PEDV/Belgorod/dom/2008			MF577027.1
165	Miniopterus schreibersii bat coronavirus 1-related isolate 161454	2018	Miniopterus schreibersii	MN611524.1
166	Alphacoronavirus sp. strain VZ_AlphaCoV_16715_47_c2	2014	Scotophilus kuhlii	MH687942.1
167	Bat coronavirus 1A			NC_010437.1
168	Alphacoronavirus sp. strain VZ_AlphaCoV_16715_39_c1	2014	Scotophilus kuhlii	MH687938.1
169	NL63-related bat coronavirus strain BtKYNL63-9a	2010	Triaenops afer	NC_032107.1
170	Alphacoronavirus sp. strain VZ_AlphaCoV_20745_6	2015	Scotophilus kuhlii	MH687966.1
171	Transmissible gastroenteritis virus strain TGEV/USA/Illinois139/2006	2006		KX900396.1
172	Feline coronavirus UU21	2007	Feliformia	HQ012369.1
173	Ferret coronavirus isolate FRCoV-NL-2010	2010	Mustela putorius	NC_030292.1
174	NL63-related bat coronavirus strain BtKYNL63-15	2008	Triaenops afer	KY073746.1
175	Bat coronavirus 1B strain AFCD307			EU420137.1
176	Feline coronavirus UU16	2007	Felis catus	FJ938058.1
177	Hipposideros bat coronavirus HKU10 isolate	2010	Chiroptera	JQ989272.1

	TLC1343A			
178	Rousettus bat coronavirus HKU10 isolate 175A	2005	Chiroptera	JQ989271.1
179	Hipposideros bat coronavirus HKU10 isolate LSH5A	2005	Chiroptera	JQ989269.1
180	Rousettus bat coronavirus HKU10	2005	Chiroptera	NC_018871.1
181	Feline coronavirus strain HF1902	2019	Felis catus	MT444152.1
182	Hipposideros pomona bat coronavirus HKU10-related isolate 160942	2018	Hipposideros pomona	MN611523.1
183	Transmissible gastroenteritis virus strain TGEV/USA/Minnesota138/2006	2006		KX900395.1
184	Ferret coronavirus genomic RNA, strain: ferret063	2016	Mustela putorius furo	LC215871.1
185	Ferret coronavirus genomic RNA, strain: FRCoV4370	2013	Mustela putorius furo	LC119077.1
186	Transmissible gastroenteritis virus strain H16	1973	Sus scrofa	FJ755618.2
187	Feline coronavirus isolate Cat 1 Karlslunde	2015	Felis catus	KX722530.1
188	Lucheng Rn rat coronavirus isolate Ruian-83	2014	Rattus norvegicus	MT820626.1
189	Transmissible gastroenteritis virus strain TGEV AHHF	2015	Sus scrofa	KX499468.1
190	Feline coronavirus strain DF-2			DQ286389.1
191	NL63-related bat coronavirus strain BtKYNL63-9b	2010	Triaenops afer	NC_048216.1
192	BtMf-AlphaCoV/HeN2013	2013	Miniopterus fuliginosus	KJ473800.1
193	Miniopterus pusillus bat coronavirus HKU8-related isolate 6610	2018	Miniopterus pusillus	MN611518.1
194	BtMf-AlphaCoV/HuB2013	2013	Miniopterus fuliginosus	KJ473798.1
195	BtMf-AlphaCoV/GD2012	2012	Miniopterus fuliginosus	KJ473797.1
196	Lucheng Rn rat coronavirus isolate Lucheng-19	2013	Rattus norvegicus	NC_032730.1
197	BtMf-AlphaCoV/FJ2012	2012	Miniopterus fuliginosus	KJ473799.1
198	Bat coronavirus HKU8			NC_010438.1
199	Minacovirus mink/NLD/2020/NT_4	2020	Neovison vison	MW248736.1
200	Feline coronavirus strain FCoV/NTU156/P/2007	2007	Felis catus	GQ152141.1
201	Mink coronavirus 1 strain MCoV1/11917-2/DK/2015	2015	Neovison vison	MN535736.1
202	Mink coronavirus 1 strain MCoV1/11918-1/DK/2015	2015	Neovison vison	MN535737.1
203	Mink coronavirus strain WD1133	1998	Neovison vison	HM245926.1
204	Alphacoronavirus Mink/China/1/2016	2016	Mustela lutreola	MF113046.1
205	Mink coronavirus strain WD1127	1998	Neovison vison	NC_023760.1
206	Canine coronavirus isolate HLJ-073	2016	Canis lupus familiaris	KY063618.2
207	Feline coronavirus isolate 27C	2013	Felis catus	KP143507.1
208	Feline coronavirus strain WSU 79-1683			JN634064.1
209	Feline infectious peritonitis virus strain DF-2		Felis catus	JQ408981.1
210	Bat alphacoronavirus isolate AMA_L_F	2015	Desmodus rotundus	MT663548.1
211	Coronavirus BtRs-AlphaCoV/YN2018	2017	Cynopterus sphinx	MK211373.1
212	Feline coronavirus strain Felis catus/NLD/UU88/2010	2010	Felis catus	KF530123.1
213	Feline coronavirus UU3	1998	Felis catus	FJ938061.1
214	Feline Alphacoronavirus 1 strain FCoV-SB22	2015	Felis catus	MH817484.1
215	Lucheng Rn rat coronavirus isolate Lijiang-71	2014	Eothenomys miletus	MT820627.1

216	Feline coronavirus strain FIPV 79-1146		Felis catus	DQ010921.1
217	Feline coronavirus isolate UG-FH8	2015	Felis catus	KX722529.1
218	Rhinolophus bat coronavirus HKU32 strain TLC26A	2015	Rhinolophus sinicus	MK720945.1
219	Feline coronavirus UU17	2007	Feliformia	HQ012367.1
220	Feline coronavirus UU54	2010	Felis catus	JN183883.1
221	Feline coronavirus RM	2002	Felis catus	FJ938051.1
222	Feline coronavirus UU40	2008	Felis catus	HQ392469.1
223	Feline coronavirus UU30	2008	Feliformia	HQ392472.1
224	Feline coronavirus isolate QS	2018	Felis catus	MW030108.1
225	Feline coronavirus isolate Cat3_day28_deletion	2013	Felis catus	KU215427.1
226	Feline coronavirus UU47	2010	Felis catus	JN183882.1
227	Feline coronavirus UU20	2007	Feliformia	HQ392471.1
228	Feline coronavirus UU18	2007	Feliformia	HQ012368.1
229	Feline coronavirus UU5	2007	Felis catus	FJ938056.1
230	Feline coronavirus UU19	2007	Feliformia	HQ392470.1
231	Feline coronavirus isolate Black			EU186072.1
232	Feline coronavirus isolate inoculum	2013	Felis catus	KU215419.1
233	Canine coronavirus isolate HLJ-072	2016	Canis lupus familiaris	KY063617.1
234	Feline coronavirus UU22 isolate TCVSP-ROTTIER-00022	2007	Feliformia	GU553361.1
235	Feline coronavirus UU24	2008	Feliformia	HQ012370.1
236	Feline coronavirus UU9	2007	Felis catus	FJ938062.1
237	Feline coronavirus UU4	2007	Felis catus	FJ938054.1
238	Canine coronavirus strain A76	1976	Canis lupus familiaris	JN856008.2
239	Feline coronavirus UU31	2008	Feliformia	HQ012371.1
240	Feline coronavirus UU15	2007	Felis catus	FJ938057.1
241	Feline coronavirus UU7	2007	Felis catus	FJ938053.1
242	Feline coronavirus strain FCoV C1Je			DQ848678.1
243	Canine coronavirus strain CB/05	2005	Canis lupus familiaris	KP981644.1
244	Feline coronavirus UU8	2007	Felis catus	FJ938055.1
245	Feline coronavirus isolate XXN	2018	Felis catus	MN165107.1
246	Feline coronavirus isolate Felix	2012	Felis catus	MG893511.1
247	Feline coronavirus isolate SD	2018	Felis catus	MW030110.1
248	Feline coronavirus strain ZJU1709	2017	Felis catus	MT239440.1
249	Feline coronavirus strain HLJ/DQ/2016/01	2016	Felis catus	KY292377.1
250	Feline coronavirus UU11	2007	Felis catus	FJ938052.1
251	Canine coronavirus strain 2020/7	2020	Canis lupus familiaris	MT906865.1
252	Feline coronavirus isolate 79-1146_CA	2018	Felis catus	MW030109.1
253	Feline coronavirus strain ZJU1617	2016	Felis catus	MT239439.1
254	Feline coronavirus strain DF-2 R3i		Felis catus	JQ408980.1
255	Canine coronavirus strain CCoV/NTU336/F/2008	2008	Canis lupus familiaris	GQ477367.1
256	Canine coronavirus strain TN-449		Canis lupus familiaris	JQ404410.1
257	Canine coronavirus strain 2020/15	2020	Canis lupus familiaris	MT906864.1
258	Feline coronavirus strain HLJ/HRB/2016/10	2016	Felis catus	KY566209.1
259	Canine coronavirus strain 1-71		Canis lupus familiaris	JQ404409.1
260	Alphacoronavirus 1 strain 23/03	2003		KP849472.1
261	Bat coronavirus isolate Anlong-57	2013	Myotis davidii	KY770851
262	Porcine epidemic diarrhea virus strain CV777		Sus scrofa domesticus	NC_003436
263	Transmissible gastroenteritis virus strain Purdue		Sus scrofa domesticus	NC_038861

264	Feline infectious peritonitis virus strain 79-1146		Felis catus	NC_002306
265	Camel alphacoronavirus isolate camel/Riyadh/Ry141/2015	2015	Camelus	NC_028752
