# Supplementary material for: Genomic Characterization of a Novel Alphacoronavirus Isolated from Bats, Korea, 2020
Source: Viruses. 2021 Oct 11;13(10):2041. doi: 10.3390/v13102041 (PMC8540747; doi:10.3390/v13102041)
Supplement: Supplementary file 1 [file viruses-13-02041-s001.zip › viruses-1334791-supplementary.pdf]

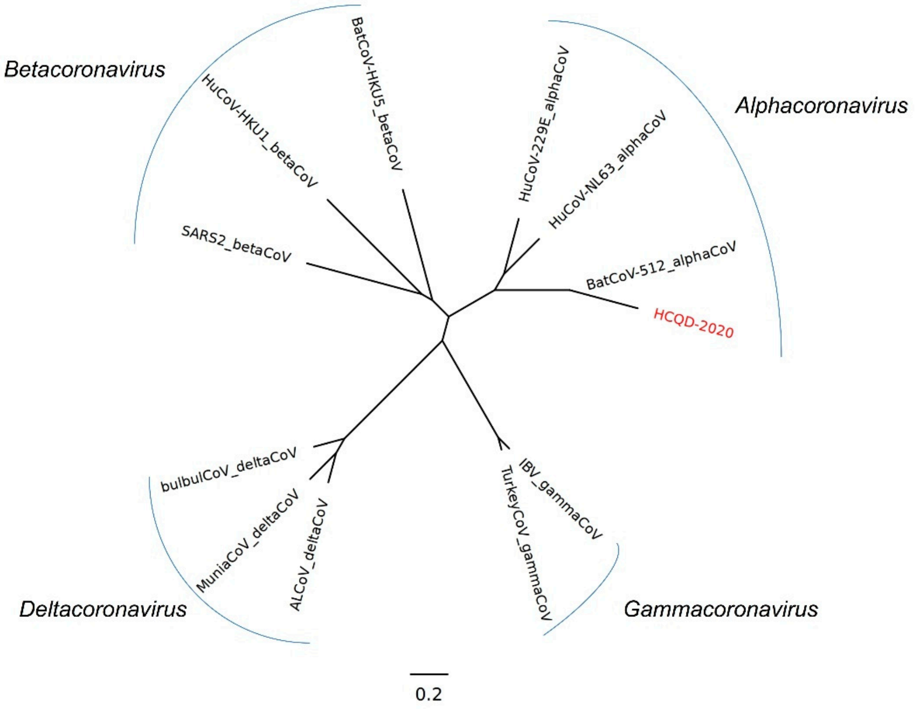

Figure S1. Initial classification of HCQD-2020 strain within subfamily *Coronavirinae*using the conserved region of Rdrp encoding fragment. It is clear that HCQD-2020 belongs to the genus *Alphacoronavirus*, highly supported by the bootstrap value. Our strain was highlighted as red.

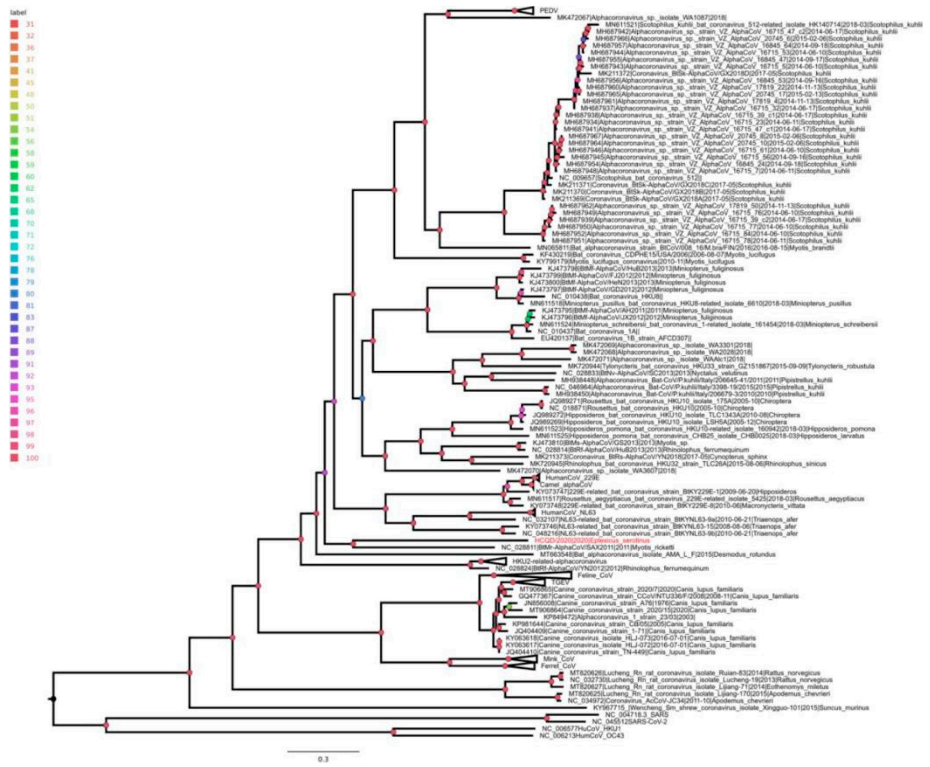

Figure S2. Phylogenetic tree construction based on whole genome sequence analysis. Results indicated that HCQD-2020 strain was distantly related with other published strains belonging to *Alphacoronavirus*. The present strain was highlighted as red.

Table S1. Information of bat samples collected in this study.

Bat species	No of samples	Collection date	Collection location
Eptescus serotinus	1	20/07/2020	Gyeongbuk
Myotis aurascens	2	03/08/2020	Gyeongbuk
Myotis petax	1	05/09/2020	Kangwon
Myotis ikonnikovi	1	05/09/2020	Kangwon
Pipstellus abranus	1	13/09/2020	Gyeongbuk

Table S2. List of primers using in this study.

Primer name	Sequence	Ta (°C)	Product size	Reference
Pan-CoF	GGTTGGGACTATCCTAAGTGTGA	55	440 bp	Poon et al., (2005)
Pan-CoR	CCATCATCAGATAGAATCATCAT A			
Alpha-F	GCACAAGTGCTTACAGAGGTTG	55	313 bp	This study
Alpha-R	CAACACCGTCATCAGACAGG			
